# *NOD-m*: a novel clinical score for predicting hearing loss in inherited metabolic disorders

**DOI:** 10.1007/s00405-025-09477-8

**Published:** 2025-06-25

**Authors:** Merve Koç Yekedüz, İlayda Kütükkiran, İlknur Sürücü Kara, Mobin Shamsaee, Feyza Nur Irem Mengüç, Engin Köse, Fatma Tuba Eminoğlu

**Affiliations:** 1 Department of Pediatric Metabolism, Ankara University Faculty of Medicine, Ankara, Türkiye; 2 Department of Anesthesiology, Critical Care and Pain Medicine, Harvard Medical School, Boston Children’s Hospital, Boston, MA USA; 3 Department of Pediatrics, Ankara University Faculty of Medicine, Ankara, Türkiye; 4 Ankara University Faculty of Medicine, Ankara, Türkiye; 5 Ankara University Rare Diseases Application and Research Center, Ankara, Türkiye

**Keywords:** Awareness, Hearing loss, Inherited metabolic disorders, Sensorineural

## Abstract

**Objectives:**

Inherited metabolic disorders (IMD) are rare genetic conditions resulting from disruptions in metabolic pathways, often leading to multisystemic complications. Hearing loss (HL) is a significant but under-recognized manifestation of IMD. Its prevalence, subtypes, timing, and systemic associations remain poorly characterized. This study aims to systematically evaluate the prevalence and characteristics of HL in a large cohort of patients with IMD, emphasizing associated clinical and laboratory findings.

**Methods:**

A retrospective review was conducted on 996 patients diagnosed with IMD between June 2012 and January 2024. Data on demographics, disease-specific features, HL subtypes, timing of diagnosis, and associated findings were analyzed. Descriptive statistics were used to summarize the findings.

**Results:**

Hearing loss was identified in 31 patients (3.1%), with lysosomal storage disorder (LSD, 30.3%) and mitochondrial disease (MD, 25.8%) being the most frequent diagnoses. Of these patients, 19 (61.2%) were male, and the mean age at the time of IMD diagnosis was 73 ± 49 months. The most common type of HL observed was sensorineural. Most common systemic findings associated with HL (HL+) included neuromotor-cognitive delay (87.1%), organomegaly (51.6%), and dysmorphism (41.9%), alongside elevated lactate levels (38.7%) and metabolic acidosis (35.5%).

The most common findings in patients with hearing loss were neuromotor-cognitive delay, organomegaly, dysmorphism, and metabolic acidosis. A composite clinical score based on these features (NOD-m) demonstrated good discriminatory performance (AUC = 0.78) for predicting hearing loss in IMD patients. The “NOD-m” code serves as a mnemonic clinical construct to aid in the early recognition of IMDs in patients with hearing loss. “NOD” reflects universal nonverbal communication, aligning with the context of auditory impairment, while “m” denotes both metabolism and metabolic acidosis—key features in the pathophysiology of these disorders.

**Conclusions:**

Hearing loss in IMD patients is frequently associated with distinct clinical and metabolic features that can guide early recognition. The NOD-m score may serve as a practical screening aid to prompt further metabolic evaluation in patients presenting with HL.

## Introduction

Inherited metabolic disorders (IMD) are rare genetic conditions associated with disruptions in metabolic pathways, leading to multisystemic complications [1]. Hearing loss (HL) is a significant yet under-recognized feature that can occur across various subtypes of IMD. However, studies investigating the incidence, onset, and systemic associations of HL in IMD are limited and predominantly based on case reports [2]. Approximately 40 inherited metabolic disorders have been identified as potentially causing hearing loss, with lysosomal storage disorders and mitochondrial diseases being the most commonly implicated [2–4].

Lysosomal storage disorders and mitochondrial diseases are both complex and multisystemic conditions. Lysosomal storage disorders result from deficiencies in lysosomal enzymes, leading to the accumulation of metabolic substrates. These disorders typically present with progressive multisystemic symptoms, such as hearing loss, dysmorphism, and organomegaly. Mitochondrial diseases, on the other hand, are characterized by defects in ATP production and commonly affect the central nervous system, skeletal muscles, and cardiovascular system. Hearing loss is frequently observed in these two significant groups. However, beyond these conditions, HL has also been reported in other inherited metabolic disorders, including biotinidase deficiency, methylmalonic acidemia, and glycogen storage disorders [2, 5, 6].

The recognition of IMD related with HL is often delayed due to the rarity and heterogeneity of these conditions, as well as the lack of predictive biomarkers. Notably, hearing loss may precede the diagnosis of an inherited metabolic disorder, presenting an often-overlooked opportunity for early intervention.

This study systematically examines the prevalence, subtypes, and onset timing of hearing loss in various inherited metabolic disorder diagnoses and highlights the associated systemic and laboratory findings.

## Methods

This is a retrospective study, and patients who were evaluated at the Department of Pediatric Metabolism, Faculty of Medicine, Ankara University between June 2012 and January 2024 were included based on their medical records. Among these, 996 patients had a confirmed diagnosis of an inherited metabolic disorder (IMD). The retrospective data review focused on socio-demographic characteristics, disease-specific features, final diagnoses, hearing loss, and related parameters.

Hearing Assessment Methods by Age Group: Hearing evaluations were conducted in accordance with age-appropriate clinical standards. For infants aged 0–6 months, the Auditory Brainstem Response (ABR) test, an objective method that does not require patient cooperation, was used. Auditory brainstem responses were mainly evaluated using click stimuli. Between 6 months and 2.5 years of age, ABR remained the primary assessment method, and when behavioral cooperation was feasible, it was complemented by Visual Reinforcement Audiometry (VRA). In children aged 2.5–5 years, play audiometry, a semi-objective behavioral method, was implemented. For children aged 5 years and older, pure tone audiometry was performed, as children in this age group can typically provide reliable verbal or motor feedback.

All patients were screened using a consistent protocol appropriate for their age; however, some patients residing outside of the capital city opted to undergo hearing assessments in their local healthcare facilities. Tympanometry was routinely performed in patients with suspected conductive hearing loss to exclude otitis media with effusion, as part of the standard audiological assessment protocol. Due to the retrospective design of the study, auditory neuropathy spectrum disorder (ANSD) could not be systematically excluded in all patients; however, no clinical or electrophysiological findings suggestive of ANSD were documented in the available records.

For statistical analysis, descriptive statistics were utilized, including measures such as mean, median, standard deviation, percentiles, minimum, and maximum values, to comprehensively summarize the data. Categorical variables were compared between patients with and without hearing loss (HL) using Fisher’s exact test due to the small sample size in the HL-positive group. The test was applied to assess whether the distribution of specific clinical features—including neuromotor delay, organomegaly, dysmorphic features, and metabolic acidosis—differed significantly between the two groups. To evaluate the ability of the selected clinical features to distinguish between patients with and without HL, we performed a receiver operating characteristic (ROC) analysis. The area under the ROC curve (AUC) was calculated to quantify the overall discriminative performance of the model. An AUC value of 0.5 indicates no discrimination (equivalent to random chance), whereas a value of 1.0 reflects perfect discrimination. The data were analyzed using the SPSS program, and patient information was extracted from their medical records. Ethical approval for this study was obtained from the local ethics committee of Ankara University Faculty of Medicine (Ethical approval number: İ02-175-25, Date: March 06, 2025).

## Results

Among the 996 patients diagnosed with IMDs, 31 patients (3.1%) were identified to have hearing loss (HL). Of these patients, 19 (61.2%) were male, and 12 (38.7%) were female. A history of consanguinity was present in 13 patients (41.9%). Twelve patients (38.7%) had siblings diagnosed with IMDs, and 9 patients (29.0%) had siblings with hearing loss. A total of 24 patients (77.4%) were born at term. The median birth weight was 3,150 g [2,500–3,400]. The median age of diagnosis for metabolic disorders was 36 months [12–96] (Table 1).

**Table 1:** Baseline characteristics of IMD patients with hearing loss

Characteristic	Category	*n* (%)
Sex, n (%)	Female	12 (38.7)
	Male	19 (61.2)
Consanguinity n (%)	No	18 (58.0)
	Yes	13 (41.9)
Sibling with Hearing Loss n (%)	No	22 (70.9)
	Yes	9 (29.0)
Sibling with a Metabolic Disease n (%)	No	19 (61.2)
	Yes	12 (38.7)
Gestational Age n (%)	Preterm	7 (22.6)
	Term	24 (77.4)
Birth Weight (grams)	Mean (SD)	3,152.85 (616.5)
	Median	3,150
	25th-75th percentile	2,500-3,400
	Min-max	1,300-3,750
Age of Diagnosis for Metabolic Disease (Months)	Mean (SD)	73 (49)
	Median	36
	25th-75th percentile	12–96
	Min-max	1-204
Survival Status n (%)	Alive	31 (100.0)

**Fig. 1:**
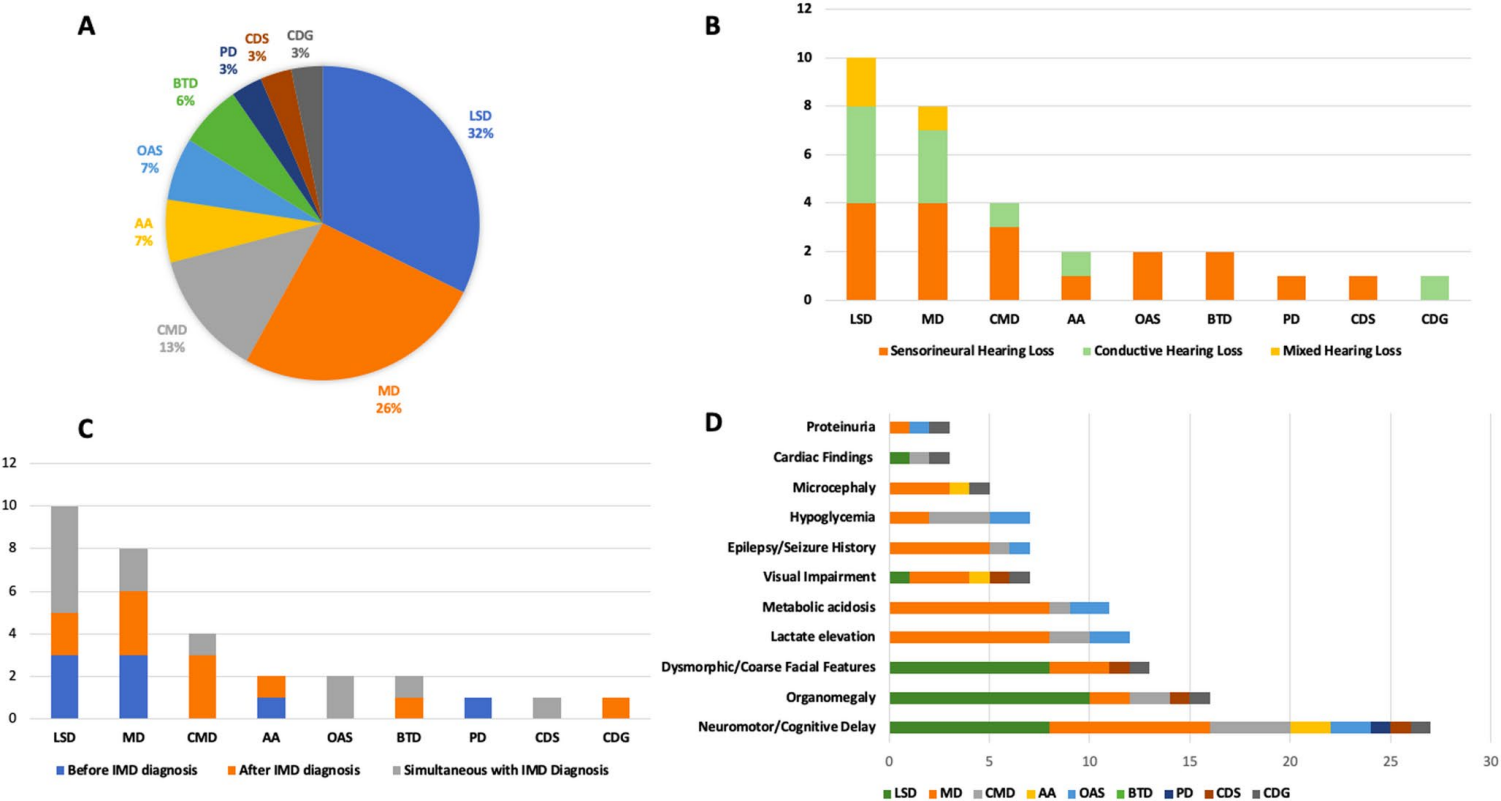
**(A)** Distribution of IMD Groups with Hearing Loss (HL is most common in LSD and MD); **(B)** Distribution of Hearing Loss Types in IMD Patients (The most frequent HL type is sensorineural); **(C)** Timing of Hearing Loss Identification in IMD Patients (HL is usually diagnosed after IMD); **(D)** Other Systemic Symptoms and Clinical-Laboratory Findings in IMD Patients with Hearing Loss (Most common clinical findings: neuromotor delay, organomegaly, dysmorphism. Most common lab finding: metabolic lactic acidosis). Abbreviations: AA: Amino Acid Metabolism Disorders, BTD: Biotinidase Deficiency, CDG: Congenital Glycosylation Defect, CDS: Chanarin-Dorfman Syndrome (Neutral Lipid Storage), CMD: Carbohydrate Metabolism Disorders, LSD: Lysosomal Storage Diseases, MD: Mitochondrial Diseases, OAs: Organic Acidemias, PD: Peroxisomal Disorders

Among the patients with hearing loss, 10 (32%) had lysosomal storage disorders, of which half were diagnosed with alpha-mannosidosis. Eight patients (25.8%) had mitochondrial diseases, and 4 (12.9%) had carbohydrate metabolism disorders (Fig. 1; Table 2).

**Table 2:** Diagnosis groups, types of hearing loss, timing of detection and hearing aids usage in IMD patients

Diagnosis Group	*n* (%)	Types of Hearing Loss, *n* (%)			Timing of Hearing Loss Detection, *n* (%)			Hearing Aids
		Sensorineural	Conductive	Mixed	Before IMD Diagnosis	After IMD Diagnosis	Simultaneous with IMD Diagnosis	Patients Using Hearing Aids
**Lysosomal Storage Diseases**	**10 (30.3)**	**4 (40.0)**	**4 (40.0)**	**2 (20.0)**	**3 (30.0)**	**2 (20.0)**	**5 (50.0)**	**4 (40.0)**
• Alpha Mannosidosis	5 (16.1)	2 (40.0)	2 (40.0)	1 (20.0)	3 (60.0)	-	2 (40.0)	3 (60.0)
• Mucopolysaccharidosis	3 (9.6)	1 (33.3)	1 (33.3)	1 (33.3)	-	-	3 (100.0)	1 (33.3)
• Gaucher Disease	2 (6.4)	1 (50.0)	-	1 (50.0)	-	2 (100.0)	-	-
**Mitochondrial Diseases**	**8 (25.8)**	**4 (50.0)**	**3 (37.5)**	**1 (12.5)**	**3 (37.5)**	**3 (37.5)**	**2 (25.0)**	**5 (55.5)**
**Carbohydrate Metabolism Disorders**	**4 (12.9)**	**3 (75.0)**	**1 (25.0)**	**0 (0.0)**	**0 (0.0)**	**3 (75.0)**	**1 (25.0)**	**1 (25.0)**
• Pompe Disease	1 (3.2)	1 (100.0)	-	-	-	1 (100.0)	-	-
• Glycogen Storage Disease Type 1a	1 (3.2)	1 (100.0)	-	-	-	1 (100.0)	-	1 (100.0)
• Glycogen Storage Disease Type 1b	1 (3.2)	-	1 (100.0)	-	-	1 (100.0)	-	-
• Galactosemia	1 (3.2)	-	-	1 (100.0)	-	-	1 (100.0)	-
**Amino Acid Metabolism Disorders**	**2 (6.4)**	**1 (50.0)**	**1 (50.0)**	**0 (0.0)**	**1 (50.0)**	**1 (50.0)**	**0 (0.0)**	**1 (50.0)**
• PTPS deficiency	1 (3.2)	-	1 (100.0)	-	1 (100.0)	-	-	-
• Prolidase Deficiency	1 (3.2)	1 (100.0)	-	-	-	1 (100.0)	-	1 (100.0)
**Organic Acidemias**	**2 (6.4)**	**2 (100.0)**	**0 (0.0)**	**0 (0.0)**	**0 (0.0)**	**0 (0.0)**	**2 (100.0)**	**0 (0.0)**
• Methylmalonic Acidemia	2 (6.4)	2 (100.0)	-	-	-	-	2 (100.0)	-
**Biotinidase Deficiency**	**2 (6.4)**	**2 (100.0)**	**0 (0.0)**	**0 (0.0)**	**0 (0.0)**	**1 (50.0)**	**1 (50.0)**	**0 (0.0)**
**Peroxisomal Disorders**	**1 (3.2)**	**1 (100.0)**	**0 (0.0)**	**0 (0.0)**	**1 (100.0)**	**0 (0.0)**	**0 (0.0)**	**0 (0.0)**
• Adrenoleukodystrophy	1 (3.2)	1 (100.0)	-	-	1 (100.0)	-	-	-
**Chanarin-Dorfman Syndrome**	**1 (3.2)**	**1 (100.0)**	**0 (0.0)**	**0 (0.0)**	**0 (0.0)**	**0 (0.0)**	**1 (100.0)**	**0 (0.0)**
**Congenital Glycosylation Defect**	**1 (3.2)**	**0 (0.0)**	**1 (100.0)**	**0 (0.0)**	**0 (0.0)**	**1 (100.0)**	**0 (0.0)**	**0 (0.0)**

*PTPS: 6-pyruvoyl-tetrahydropterin synthase

40% of patients with lysosomal storage disorders had sensorineural hearing loss, 40% had conductive hearing loss, and 20% had mixed hearing loss. Half of these patients were diagnosed with hearing loss simultaneously with IMD diagnosis. Among patients with alpha-mannosidosis, 60% were diagnosed with hearing loss prior to their IMD diagnosis, while 40% were diagnosed concurrently. No patients in the alpha-mannosidosis group were identified with hearing loss prior to their IMD diagnosis. In the mucopolysaccharidosis group, sensorineural, conductive, and mixed types of hearing loss were observed equally (33.3%), with all cases diagnosed after the IMD diagnosis. Hearing loss in all patients with Gaucher disease was diagnosed after the IMD diagnosis. Among mitochondrial diseases, 50% of patients had sensorineural hearing loss. The timing of hearing loss diagnosis was evenly distributed, occurring both before and after IMD diagnosis (37.5%). In the carbohydrate metabolism disorder group, 75% of patients had sensorineural hearing loss, with hearing loss diagnosed in 75% of cases after the IMD diagnosis. In the amino acid metabolism disorder group, PTPS deficiency and prolidase deficiency were diagnosed, associated with conductive and sensorineural hearing loss, respectively. All patients with organic acidemias had sensorineural hearing loss, diagnosed simultaneously with their IMD. In the peroxisomal disorder group, one patient with adrenoleukodystrophy was diagnosed with sensorineural hearing loss prior to their IMD diagnosis. One patient with CDG was diagnosed with conductive hearing loss after their IMD diagnosis (Fig. 1; Table 2). The most frequently observed audiometric configurations were flat and sloping, consistent with sensorineural hearing loss patterns.

The most common associated findings in IMD patients with hearing loss included neuromotor and cognitive delay (*n* = 27, 87.1%), organomegaly (*n* = 16, 51.61%), and dysmorphic/coarse facial features (*n* = 13, 41.94%) (Fig. 1). Neuromotor and cognitive delay were most frequently observed in lysosomal storage disorders (*n* = 8, 29.6%) and mitochondrial diseases (*n* = 8, 29.6%). Organomegaly was most commonly identified in lysosomal storage disorders (*n* = 10, 62.5%).

Epilepsy (*n* = 5, 71.4%), microcephaly (*n* = 3, 60%), and visual impairment (*n* = 3, 42.8%) were most frequently observed in patients with mitochondrial diseases. The most common laboratory findings were elevated lactate levels (*n* = 12, 38.71%) and metabolic acidosis (*n* = 11, 35.48%). Hypoglycemia was most frequently seen in patients with carbohydrate metabolism disorders (*n* = 3, 42.8%) (Fig. 1).

***NOD-m Code***: *N: Neuromotor-cognitive delay*,* O: Organomegaly*,* D: Dysmorphism*,* m: Metabolic Lactic Acidosis*.

**Fig. 2:**
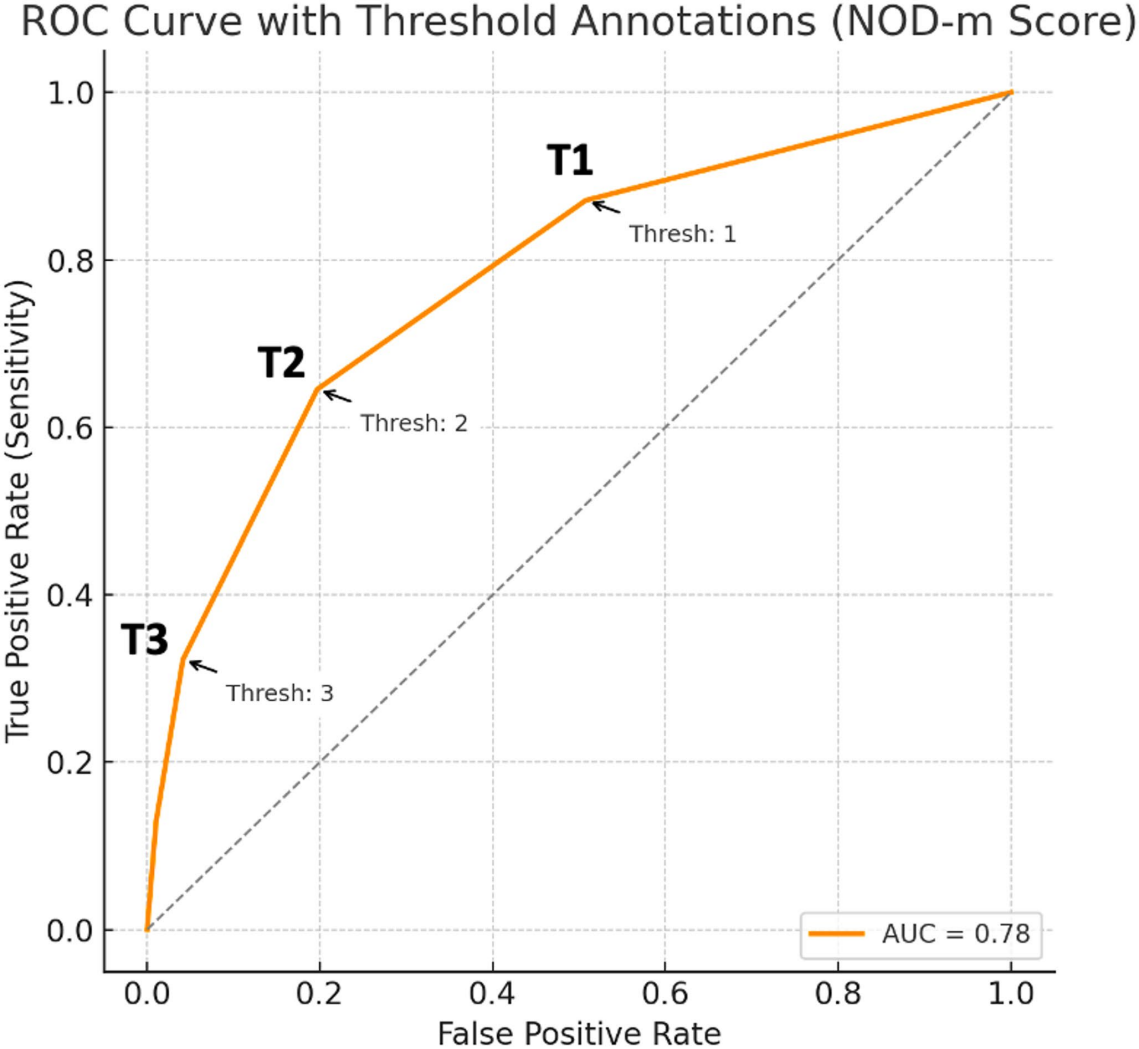
ROC curve showing the diagnostic performance of the NOD-m score in predicting hearing loss, with threshold annotations. **Threshold = 1 (T1)**: Patients with one positive NOD-m feature are considered at risk for HL. *High sensitivity (87.1%)*,* but low specificity (49.2%).***Threshold = 2 (T2)**: Patients with two positive NOD-m features are considered at risk. *Balanced performance: Sensitivity of 64.5% and specificity of 80.3%.***Threshold = 3 (T3)**: Patients with three or more positive NOD-m features. *High specificity (95.9%)*,* but lower sensitivity (32.3%)*

Among the 996 patients evaluated, neuromotor delay (87.1% vs. 42.8%, *p* < 0.001), organomegaly (51.6% vs. 32.3%, *p* = 0.0318), dysmorphic features (41.9% vs. 12.0%, *p* < 0.001), and metabolic acidosis (35.5% vs. 19.8%, *p* = 0.0408) were significantly more frequent in patients with hearing loss (HL+) compared to those without (HL–), based on Fisher’s exact test (Table 3). A receiver operating characteristic (ROC) analysis was performed using these four features, collectively referred to as the NOD-m score. The area under the curve (AUC) was 0.78, indicating good discriminatory power in distinguishing HL + from HL – patients. Three NOD-m score thresholds were evaluated: **Threshold = 1** (one feature present): yielded a high sensitivity of 87.1% but low specificity of 49.2%. **Threshold = 2** (two features): achieved a balanced performance with 64.5% sensitivity and 80.3% specificity. **Threshold = 3** (three or more features): resulted in higher specificity (95.9%) but lower sensitivity (32.3%) (Fig. 2). These findings suggest that increasing the number of NOD-m features improves specificity at the expense of sensitivity.

**Table 3:** Distribution of NOD-m features in IMD-patients with hearing loss and without hearing loss

Feature, *n* (%)	HL (+) *n* = 31	HL (–) *n* = 965	*p*-value
**N** (neuromotor delay)	27 (87.1)	413 (42.8)	< 0.001
**O** (organomegaly)	16 (51.6)	312 (32.3)	0.0318
**D** (dysmorphism)	13 (41.9)	116 (12.0)	< 0.001
**m** (metabolic acidosis)	11 (35.5)	191 (19.8)	0.0408

HL (+): patients with hearing loss, HL(-): patients without hearing loss

The term “NOD” was deliberately selected not only as an acronym but also as a symbolic reference to nonverbal communication and mutual understanding—concepts that are particularly salient in the context of hearing loss. Universally recognized as a gesture denoting agreement and acknowledgment, the “nod” also reflects the silent yet profound ways in which individuals with hearing impairment often engage with the world. Within this framework, “N” denotes neuromotor-cognitive delay, “O” represents organomegaly, and “D” signifies dysmorphism—collectively the most prevalent clinical phenotypes identified among IMD patients with hearing loss. The appended “m” serves a dual function: it denotes metabolic lactic acidosis, the most frequently observed laboratory abnormality, while simultaneously invoking the broader domain of “metabolism,” thereby reinforcing both clinical relevance and mnemonic utility. This composite term, NOD-m, thus encapsulates a constellation of multisystemic indicators that may guide clinicians toward the early recognition of co-occurrence of IMD and HL.

## Discussion

Our study is a comprehensive investigation into the frequency, subtypes, timing, and associated findings of hearing loss (HL) in patients diagnosed with various IMD. It has been identified that the most common IMD associated with hearing loss are lysosomal storage disorders and mitochondrial diseases. The most frequently observed systemic findings accompanying hearing loss were neuromotor-cognitive delay (N), organomegaly (O), and dysmorphism (D), while the most commonly associated laboratory finding was metabolic lactic acidosis (m). The study highlights the importance of considering inherited metabolic disorders when HL is accompanied by NOD-m findings.

Inherited metabolic disorders encompass approximately 1,450 different disease groups, with over 80% of them being inherited in an autosomal recessive manner [1, 7, 8]. Consequently, consanguinity and a family history of similar findings, including HL, should raise suspicion for IMD. In countries like Türkiye, where the rate of consanguineous marriages is as high as 24%, these conditions are more prevalent, emphasizing the critical importance of heightened awareness of multisystemic findings across all clinical departments. In our study, nearly half of the cases reported consanguinity, and more than a quarter had siblings with HL. Therefore, in the presence of consanguinity and a sibling with HL, clinicians should maintain a high index of suspicion for genetically based disorders, particularly IMDs.

Studies on the association of hearing loss with inherited metabolic disorders are predominantly case-based, and large-scale incidence studies encompassing heterogeneous diagnoses are very limited. Approximately 40 inherited metabolic disorders have been reported to be associated with HL [2]. The most common diagnoses associated with hearing loss include diseases of complex molecules, such as alpha-mannosidosis, Niemann-Pick type C, and congenital glycosylation disorders, as well as disorders of energy metabolism [2, 3, 9–12]. The temporal relationship between HL and the diagnosis of IMD may vary depending on the underlying disease pathophysiology and the timing of clinical evaluation. In certain IMD subtypes, such as lysosomal storage disorders like alpha-mannosidosis and mitochondrial disorders, early cochlear involvement may result in HL being one of the initial manifestations, leading to its detection prior to the IMD diagnosis. In contrast, in other metabolic conditions such as biotinidase deficiency, the disease may follow a more subtle and protracted course, with HL emerging later or remaining subclinical for an extended period. Additionally, the retrospective nature of the study may limit the precise determination of symptom onset in some cases. In line with the literature, our study also determined that hearing loss is most frequently associated with lysosomal storage disorders and mitochondrial diseases. Notably, these two primary diagnostic groups represent more than half of our population with hearing loss. This finding underscores the need to develop a distinct and focused perspective on hearing loss in these two main conditions.

Lysosomal storage disorders are a group of genetically IMD characterized by the accumulation of metabolic substrates within lysosomes due to deficiencies or dysfunctions of lysosomal enzymes. These disorders often manifest with progressive multisystemic symptoms over the years [13]. Among lysosomal storage disorders, alpha-mannosidosis is most strongly associated with hearing loss, with nearly 100% affected patients presenting with hearing loss prior to the diagnosis of the IMD [2, 3]. In our study, patients with alpha-mannosidosis accounted for half of the lysosomal storage disorder cases associated with HL, and all were diagnosed with HL either before or during the diagnostic evaluation for the IMD. Consistent with the literature, sensorineural, conductive, and mixed types of hearing loss were identified in these patients. Dysmorphic features and organomegaly, other significant multisystemic findings in lysosomal storage disorders [14], often prompted suspicion of IMD in patients presenting with hearing loss. The strong and consistent association of alpha-mannosidosis with HL, often detected prior to the diagnosis of an IMD, highlights the critical importance of clinicians remaining vigilant for accompanying warning signs such as organomegaly and dysmorphy. Early identification of these patients is invaluable, as therapeutic options such as velmanase alfa enzyme replacement therapy (ERT) and hematopoietic stem cell transplantation (HSCT) offer promising outcomes [15].

The second major group in our study was mitochondrial diseases. Mitochondrial diseases are a group of disorders characterized by multisystemic involvement, severe lactic acidosis, skeletal and cardiac muscle involvement due to ATP deficiency, severe hypotonia, psychomotor retardation, failure to thrive, feeding problems, muscular atrophy, dystonia, and predominantly neurological symptoms, often with a poor prognosis. There is no definitive pattern in the onset time or type of hearing loss in mitochondrial diseases. Mitochondrial diseases may present with fatal outcomes during the neonatal period or have delayed diagnoses extending into adulthood [12, 16, 17]. Hearing loss has frequently been described as part of the phenotypic spectrum of mitochondrial diseases [18]. It is such a commonly reported finding that prevalence rates as high as 75–91% have been observed in some mitochondrial diseases series [2, 4]. In our study, diversity was observed in the timing and type of hearing loss in mitochondrial diseases. Neuromotor-cognitive delay and metabolic lactic acidosis were identified as significant warning signs accompanying hearing loss in this diagnostic group. This aligns closely with the heterogeneous nature and variable onset of mitochondrial diseases. Pathogenic defects in energy metabolism have been identified in over 400 genes to date. Only a small fraction of these genes are located in mitochondrial DNA, with the associated diseases being maternally inherited or sporadic. The remaining disease-associated genes are encoded in nuclear DNA and cause disorders inherited according to Mendelian principles, predominantly autosomal recessive [19]. Therefore, in this second major diagnostic group linked to hearing loss, family history and pedigree information, irrespective of sex, can provide critical diagnostic clues.

The association between carbohydrate metabolism disorders and hearing loss has been documented through case reports and small series [2]. Pompe disease is both a carbohydrate metabolism disorder and a lysosomal storage disorder caused by a deficiency of the acid α-glucosidase enzyme. It is clinically characterized by muscle weakness and cardiac problems. Hearing loss associated with Pompe disease has been rarely reported [20]. Since the initial symptom is usually related to muscle involvement, hearing loss is typically identified after IMD diagnosis. Knockout mouse models have demonstrated that glycogen accumulation in inner and outer hair cells, supporting cells, stria vascularis, and ganglion spiral neurons is associated with sensorineural HL [21]. In our study, one patient with Pompe disease was diagnosed with sensorineural HL after the diagnosis of the IMD. Late-onset Pompe disease often presents with subtle symptoms, leading to delayed diagnosis. In contrast, patients with infantile-onset Pompe disease typically exhibit severe muscular manifestations before hearing loss becomes apparent [22, 23].

Hepatic glycogen storage disorders, a subgroup of carbohydrate metabolism disorders, are primarily characterized by hypoglycemia, hyperlactatemia, hyperuricemia, and hepatomegaly. These patients require frequent feeding both during the day and at night. Severe hypoglycemic attacks can lead to sensorineural hearing loss, while nocturnal feeding is a risk factor for the development of gastroesophageal reflux (GER), which in turn can cause chronic otitis media and, consequently, conductive hearing loss [6]. In our study, two patients with glycogen storage disorders (types 1a and 1b) developed sensorineural and conductive hearing loss, respectively, as complications after the diagnosis of their IMD. As observed in cases of conductive hearing loss associated with glycogen storage disorders, hearing loss can be a preventable complication in certain patients. Evaluating patients for the risk of GER and transitioning to percutaneous endoscopic gastrostomy tube feeding for those at risk of chronic otitis media are proactive measures that can be taken to mitigate the risk of hearing loss. However, as an invasive method, its benefits and risks must be carefully weighed [24].

Another group with identified HL is methylmalonic acidemia, an acute intoxication-type inherited metabolic disorder. It is characterized by the accumulation of methylmalonic acid resulting from a deficiency in the methylmalonyl-CoA mutase enzyme [25]. Patients may present shortly after birth with acute deterioration, metabolic acidosis, and hyperammonemia or later in life with a more heterogeneous clinical picture, often resulting in early death or severe neurological damage [26]. Although HL is infrequently reported in methylmalonic acidemia, our study identified sensorineural HL in these patients during routine screening. This is consistent with limited studies suggesting that elevated levels of toxic organic acids, such as methylmalonic acid and propionic acid, disrupt auditory pathway function by interfering with potassium channels [27]. This diagnostic group underscores the importance of thoroughly evaluating systemic involvement during routine follow-ups of IMDs, even in cases where HL is reported as a rare complication.

Another critical diagnostic group worth emphasizing is biotinidase deficiency. Biotinidase deficiency is an autosomal recessive neurocutaneous IMD. Untreated patients can present with a range of severe neurological and dermatological symptoms, including seizures, hypotonia, feeding difficulties, developmental delay, hearing loss, optic atrophy, ataxia, alopecia, and skin rash [28, 29]. In Turkey, biotinidase deficiency is one of only two IMDs included in the national newborn screening program (the other being phenylketonuria), enabling early diagnosis and prompt treatment [30]. Administering biotin (vitamin H) supplementation to newborns as soon as the deficiency is identified is the most crucial intervention to prevent hearing loss [31]. In the literature, the prevalence of HL in patients with biotinidase deficiency is reported to be as high as 76% [32–34]. However, in our cohort of 110 biotinidase deficiency patients, only two cases of hearing loss were observed, a rate significantly below the reported prevalence. The key factor behind this discrepancy is likely the effectiveness of the newborn screening program and early treatment, underscoring the critical role of expanded newborn screening in preventing complications in IMDs.

Our study screened nearly one thousand patients with inherited metabolic disorders (IMDs) to identify those with hearing loss (HL). Although the heterogeneity of IMD diagnoses may limit generalizability, the inclusion of a broad diagnostic spectrum provides a valuable and comprehensive perspective. While we presented disease-specific findings within distinct subgroups, the study collectively offers an integrative understanding of IMD-associated hearing loss. Importantly, the consistent emergence of NOD-m features across different diagnoses provides clinically meaningful guidance for the early recognition and screening of patients with suspected IMDs. The emphasis on these findings raising suspicion of IMD in cases of HL stems from the fact that more than half of our study population is comprised of lysosomal storage disorders and mitochondrial diseases. As discussed earlier, these two primary diagnostic groups present with the most significant clinical and laboratory findings associated with HL. For clinicians encountering HL, recognizing and considering these associated findings (NOD-m) is crucial for raising suspicion of IMD, as early diagnosis and timely treatment are the cornerstone for improved outcomes. Similarly, patients diagnosed with IMD should undergo routine auditory evaluations to ensure early detection and management of HL, as evidenced by our population, where conditions exist in which the development of HL can be prevented through simple vitamin supplementation (biotin) or more rigorous treatments such as ERT or HSCT.

This study has several limitations that should be acknowledged. First, its retrospective design may have introduced biases related to documentation quality and limited the ability to determine the precise temporal relationship between symptom onset and diagnosis. Additionally, auditory neuropathy spectrum disorder (ANSD) could not be systematically ruled out due to the unavailability of complete electrophysiological data in all patients. The sample size for some IMD subtypes was limited, which may affect the generalizability of subtype-specific findings. Future prospective, multicenter studies with larger and more diverse IMD populations are needed to validate the NOD-m score, assess its predictive performance in real-time clinical settings, and explore the underlying pathophysiological mechanisms linking specific IMDs with hearing loss.

Importantly, to our knowledge, this is the first study in the literature to provide a prediction model for hearing loss based on such clinical markers in patients with IMD. The NOD-m code, derived from these features, demonstrated good discriminatory ability (AUC = 0.78) and offers a practical, low-cost approach for early identification of patients at risk of HL. As such, our findings may serve as a foundation for prospective validation studies and for the development of clinical screening tools in pediatric metabolic and genetic evaluation settings.

As a conclusion, our study underscores the critical importance of recognizing IMD as potential underlying causes of hearing loss, particularly when accompanied by systemic findings (such as NOD-m). The prominent representation of lysosomal storage disorders and mitochondrial diseases among patients with hearing loss further highlights the need for heightened clinical awareness and the integration of routine auditory evaluations into the follow-up of IMD patients. Early identification and intervention are key, as demonstrated by the potential to prevent hearing loss through timely treatments. Expanding newborn screening programs and fostering a multidisciplinary approach are essential steps to improving outcomes in this patient population. Further large-scale studies are warranted to validate these findings and guide evidence-based strategies for early diagnosis and management.
